# Validation of a patient satisfaction questionnaire for anemia treatment, the PSQ-An

**DOI:** 10.1186/1477-7525-4-28

**Published:** 2006-05-03

**Authors:** Robert J Nordyke, Chih-Hung Chang, Chiun-Fang Chiou, Joel F Wallace, Bin Yao, Lee S Schwartzberg

**Affiliations:** 1 Cerner Health Insights, 9100 Wilshire Blvd. Ste. 655E, Beverly Hills, CA 90290, USA; 2 UCLA School of Public Health, Los Angeles, CA, USA; 3 Northwestern University Feinberg School of Medicine, Chicago, IL, USA; 4 Amgen, Thousand Oaks, CA, USA; 5 The West Clinic, Memphis, TN, USA

## Abstract

**Background:**

Treating anemia associated with chemotherapy and many cancers is often necessary. However, patient satisfaction with anemia treatment is limited by the lack of validated instruments. We developed and validated a new treatment-specific patient satisfaction instrument: the Patient Satisfaction Questionnaire for Anemia Treatment (PSQ-An). Treatment burden and overall satisfaction scales were designed for ease of use in clinical practice.

**Methods:**

312 cancer patients (141 breast, 69 gynecological, and 102 non-small cell lung) were targeted to complete the PSQ-An at 4 week intervals. Data from weeks 5 and 9 were analyzed. Patients also completed the MOS SF-36 Global Health assessment and questions concerning resources devoted to anemia treatment. Item reduction used endorsement rates, floor/ceiling effects, and item-item correlations. Factor analysis identified meaningful subscales. Test-retest reliability was assessed. Construct validity was tested, using Pearson's correlations, by comparing subscale scores to Global Health, hemoglobin levels, and resources devoted to anemia treatment.

**Results:**

The overall response rate was 92.9% (264/284) at week 5. Most (84.2%) of the patients were female, and the mean (SD) age was 60.2 (± 11.8) years. Two distinct subscales were identified measuring treatment burden (7 items) and overall satisfaction (2 items). Test-retest reliability was examined (ICC: 0.45–0.67); both were internally consistent (alpha = 0.83). Both subscales exhibited convergent and divergent validity with independent measures of health. ANOVA results indicated that the PSQ-An Satisfaction subscale discriminated between 5 levels of MOS SF-36 Global Health (*P *= 0.006).

**Conclusion:**

The PSQ-An is a validated, treatment-specific instrument for measuring satisfaction with anemia treatment for cancer patients. PSQ-An subscales reflect the burden of injection anemia treatment on cancer patients and their assessment of the overall treatment value.

## Background

Anemia and subsequent fatigue have long been recognized as common side effects of cancer itself and its treatments [1,2]. Depending on the type and stage of cancer and the definition of anemia, the prevalence of anemia among cancer patients may be quite high. Rates of anemia (hemoglobin [Hb] <12.0 g/dL) have been reported to be 41% to 82% among breast cancer patients [3-5], 48% to 84% in lung cancer patients [3,5], and 26% to 85% among patients with ovarian or cervical cancer [3,5,6].

Furthermore, the presence of anemia is associated with decreased health-related quality of life (HRQL). Holzner [7] found a correlation between HRQL and Hb levels in mildly anemic patients (Hb >10.0 g/dL). Lind et al [8] reported a significant correlation between Hb levels and HRQL scores. At the same time, anemia treatments themselves have shown mostly positive results in improving HRQL in patients responding to treatment [9-14].

Satisfaction with treatment is an important, but poorly studied, aspect of the quality of treatment in supportive oncology care. However, despite the high prevalence of anemia and the growing recognition of treating anemia in cancer patients, there is no assessment tool for evaluating cancer patients' satisfaction with anemia treatment. Defined as a patient-reported assessment of receiving treatment and the outcomes of treatment [15], treatment satisfaction is important for a number of reasons. Foremost is the link with compliance and adherence to treatments [16-18]. Treatment satisfaction may also be an important measure for physicians and patients when choosing appropriate treatments, especially when the options have similar efficacy. Finally, competition among providers in today's healthcare marketplace has elevated the importance of patients' assessments of the quality of their healthcare [19]. Patient satisfaction measures have been studied for general medical and pharmacy services as well as for treatment of specific conditions [20-25].

We developed and validated the Patient Satisfaction Questionnaire for Anemia Treatment (PSQ-An), a disease- and treatment-specific instrument for measuring satisfaction with anemia treatment for cancer patients. The instrument addresses the gap in treatment evaluation tools for oncology care. The PSQ-An was designed to include domains that capture patients' satisfaction with the treatment itself and to include domains pertaining directly to anemia treatment: patient's general satisfaction with treatment, convenience of treatment for patient and family/friends, patients' pain and discomfort, and financial aspects of treatment for the patient. This study reports on the development and validity testing of the scale part of the PSQ-An instrument. Since most enrolled patients were women due to inclusion criteria, this effort should be viewed as an initial validation of the tool; as with most PRO measures, further validation in other patient populations is warranted.

## Methods

### Patients

The study protocols were approved by the Institutional Review Boards of participating medical centers, and all patients provided written informed consent before any study-related procedures were performed. Patients in the 3 trials were required to have a diagnosis of breast cancer, non-small cell lung cancer (NSCLC; stage IIIb or IV), or gynecologic carcinoma of the ovary, cervix, or uterus. Additional inclusion criteria were the following: ≥ 18 years old, anemic (Hb <11 g/dL at screening), expecting to receive ≥ 8 additional weeks of chemotherapy, a Karnofsky performance scale score ≥ 50%; adequate renal function (serum creatinine concentration ≤2.0 mg/dL), adequate liver function (aspartate aminotransferase or alanine aminotransferase ≤ 2 times the upper limit of the normal range or serum bilirubin ≤ 1.5 times the upper limit of the normal range), and able to complete questionnaires. Patients were excluded from the trials if they had received a red blood cell transfusion within 4 weeks of screening, or erythropoietic therapy within 2 weeks of randomization; had inadequate iron stores (transferrin saturation < 15% and ferritin < 10 ng/L); known positive antibody response to any erythropoietic agent; known history of pure red cell aplasia, of anemia due to hematologic disorders other than chemotherapy-induced anemia, or of uncontrolled hypertension.

### Initial item development

The components of the PSQ-An were drawn from other patient satisfaction instruments [26] for other injection treatments (eg, insulin injections, growth factor injections) [27,28]. Questions from these components were selected as candidate questions for the PSQ-An if they could be modified to capture 1 of the 4 preselected domains of patient satisfaction for anemia treatment (general patient satisfaction, convenience of treatment for the patient and their family and friends, patients' pain and discomfort, and financial burden for the patient). These domains were first identified from the literature review and then selected by the study team based on their relevance to anemia treatment. Redundant questions thought to be capturing the same information as other questions were removed to decrease the size of the final questionnaire. The result was a provisional 21-item instrument comprising 2 parts: a descriptive part (11 items), which included questions about resources devoted to treatment, and a scale part (10 items), which included questions about treatment burden and overall satisfaction. The questionnaire is presented in Appendix (see Additional file 1).

### Study design

The study sample consisted of 312 adult, English-speaking patients participating in 3 randomized, multicenter trials. This sample size ensures a precision of <5% in the standard errors assuming treatment compliance rates of better than 70%. For logistical and administrative reasons, 3 identical but separate protocols were used, 1 for each tumor type (breast cancer, non-small cell lung cancer, gynecologic carcinoma) with a preplanned analysis of all individual data across studies prespecified in each protocol. Patients with breast cancer (n = 141), gynecological malignancies (n = 69), or non-small cell lung cancer (n = 102) were enrolled in the study and were randomized to treatment with darbepoetin alfa or epoetin alfa for anemia due to chemotherapy. The inclusion of patients with 3 different tumor types reduces the likelihood that treatment satisfaction responses are unique to a single population of cancer patients. Following a 1-week screening period, complete blood counts (including Hb) were measured every 2 weeks prior to dosing. In addition, the 4-week recall patient satisfaction questionnaire was administered at weeks 5, 9, 13, and 17.

### Other study measures

Karnofsky Performance Status ratings (0% – Dead to 100% – Normal, no complaints, no evidence of disease) were collected in the trial and converted to Eastern Cooperative Oncology Group (ECOG) Performance Status Ratings to reduce the number of categories with very small numbers of patients used in this analysis. The ECOG Performance Status Rating measures how cancer affects the daily living abilities of the patient [29]. The scale ranges from 0 (fully active, no restrictions) to 5 (dead), where lower scores represent better mobility.

The 1-item self-report Global Health question from the MOS SF-36 was included ("In general, would you say your health is...?") with a 5-point Likert Scale, where a higher score represented better health.

### Development of the PSQ-An

The items in the scale part of the instrument originally had response values of 0 to 4 (not at all/mildly/somewhat/moderately/extremely). Values of the 7 negatively stated questions (items 1, 3, 4, 5, 6, 7, and 8) were reverse scored, so that higher values indicate more positive satisfaction. Items were considered for deletion if they met any of the following 3 criteria: 1) missing responses greater than 10% (endorsement rate); 2) more than 50% of participants reporting either the highest or lowest score available (floor/ceiling effect); or 3) significant item-item correlations ≥ 0.70 [30]. All analyses were completed for data collected at week 5 (test-retest analyses also included data collected at week 9 to maximize available sample size).

Principal component analysis was used to identify meaningful and interpretable factors. The number of factors to retain was based on eigenvalues ≥ 1, with factor loadings serving as an indicator of the degree to which each item was associated with each factor. Items were retained in a given factor if they had a factor loading ≥ 0.40. Multi-trait scaling was carried out to evaluate item convergence within scales and item discrimination across scales. *A priori *instrument reliability criteria included: 1) item correlation ≥ 0.40 with the total questionnaire (ie, item-internal consistency) [30,31], and 2) Cronbach's alpha coefficients = 0.70 (internal consistency) [32].

Test-retest reliability or reproducibility was assessed using the intraclass correlation coefficients (ICC) [33]. Responses to the MOS SF-36 Global Health question were used to identify participants with stable health status (ie, whose responses did not change across weeks 5 through 9). ICCs were computed based on this subsample, for the 5 subscale scores at both time points. A predetermined threshold for test-retest reliability was defined as an ICC of 0.70 or greater [32,34].

Convergent and divergent validity were examined by estimating Pearson's correlation coefficient and Spearman's rank-order correlation coefficient, between subscales of the PSQ-An and the MOS SF-36 Global Health, Hb level, and measures of time devoted to treatment hypothesized to assess either similar or different constructs [35-37]. We hypothesized that the scores for the subscales of the PSQ-An measuring aspects of treatment burden would correlate more strongly with the questions relating to time devoted to treatment. Further, the satisfaction subscale of the PSQ-An was expected to have a larger correlation coefficient with the MOS SF-36 Global Health score and Hb level than with resources devoted to treatment. The above correlation coefficients with the MOS SF-36 Global Health score and Hb levels were hypothesized to be positive and those with measures of time required for treatment were expected to be negative.

Discriminant validity was assessed by relating PSQ-An subscale factor scores to 3 variables measuring different aspects of patient health: MOS SF-36 Global Health score, Hb level, and ECOG scores. Mean scores on the subscales of the PSQ-An were compared across response categories of the 3 known measures using analyses of variance (ANOVA) [38]. Responsiveness was evaluated by 1) week 5 to 9 effect sizes and 2) ANOVA on week 5 to 9 changes in each PSQ-An subscale and changes in MOS SF-36 Global Health responses. Weeks 5 and 9 were chosen as the best balance between adequate sample size due to patient drop-out in the study and time required for anemia treatments to be effective.

Statistical analyses were performed using SAS version 8.2 for UNIX (SAS Institute, Cary, NC).

## Results

### Sample characteristics

**Figure 1:**
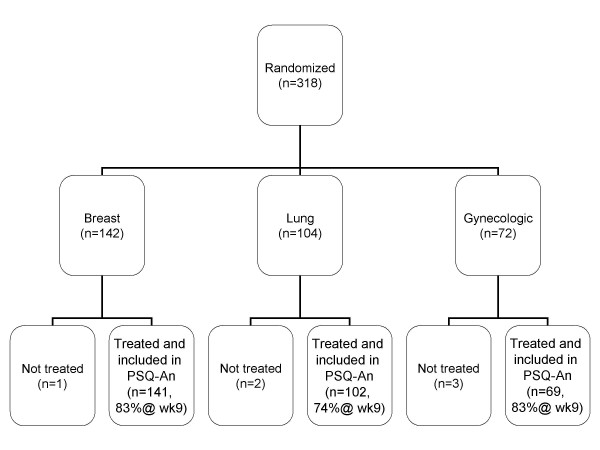
**Patient Flow Diagram**. Initial sample sizes and proportion remaining at week 9 shown.

The flow of patients initially enrolled in the study is depicted in Figure 1. Note that by week 9, only 80% of the patients remained in the study. Of the 284 patients enrolled at week 5, 264 (92.9%) participants completed the questionnaires at week 5. Table 1 summarizes the demographic and clinical characteristics of the study group classified by anemia treatment. Due to inclusion of breast and gynecological cancers, the sample was primarily female (84.2%, Table 1). Mean ages were 58.7 (11.5) and 61.7 (12.1) respectively in the darbepoetin alfa and epoetin alfa treatment groups. A total of 84.2% percent of the participants were non-Hispanic Whites. Nearly half of all participants (48.4%) had Stage IV cancer.

**Table 1:** Baseline Sample Characteristics

	**Darbepoetin alfa **(n = 157)	**Epoetin alfa **(n = 155)
Sex, n (%)		
Male	23 (15)	26 (17)
Female	134 (85)	129 (83)
Race, n (%)		
White	132 (84)	131 (85)
Black	16 (10)	11 (7)
Hispanic	3 (2)	6 (4)
Asian	6 (4)	5 (3)
Other	0 (0)	2 (2)
Age (years)		
Mean (SD)	58.7 (11.5)	61.7 (12.1)
Tumor type, n (%)		
Breast	72 (46)	69 (45)
Non Small Cell Lung (stage IIIb/IV)	51 (32)	51 (33)
Gynecologic	34 (22)	35 (23)
Stage of Disease		
I/II	41 (26)	28 (18)
III	29 (18)	27 (17)
IIIb	15 (10)	14 (9)
IV	70 (45)	81 (52)
Unknown	2 (1)	5 (3)
Karnofsky Performance Status, n (%)		
100	18 (11)	18 (12)
80, 90	104 (66)	103 (67)
60, 70	35 (22)	33 (22)
≤50	0 (0)	1 (1)
Hb (g/dL)		
Mean (SD)	10.4 (0.8)	10.4 (0.8)
Median	10.6	10.6
Hb, n (%)		
<10 g/dL	38 (24)	38 (25)
≥10 g/dL	119 (76)	117 (75)

### Item reduction

Response rates on all PSQ-An question items were greater than 90% (Table 2). Mean, standard deviation, and proportion reporting extreme values for each item are also reported in Table 2. As can be seen, 3 items exhibited ceiling effects with over 50% of responses at the highest score (difficulty receiving injection, financial burden, and likelihood of recommendation). Because the highest response marker for each item represented highest satisfaction and effectively captured potential dissatisfaction, we retained these items for further factor development.

**Table 2:** Response rate, percentage of patients choosing the lowest response marker, and percentage of patients choosing the highest response marker

**Question Item**	**Mean (SD)**	**Response rate (%)**	**% with lowest marker**	**% with highest marker**
Demands of treatment	3.14 (1.09)	92	3.2	46.5
Schedule flexibility	2.97 (1.42)	92	11.3	49.7
Difficulty in receiving every injection	3.68 (0.79)	93	1.8	75.7
Treatment-related travel interference w/daily activity	3.12 (0.98)	93	1.4	40.9
Overall inconvenience	3.27 (0.97)	93	1.8	48.6
Inconvenience to family/caregivers	3.23 (1.05)	93	2.5	49.7
Overall physical discomfort from injections	3.00 (1.02)	93	2.1	36.3
Financial burden from out-of-pocket costs	3.62 (0.75)	93	0.4	66.9
Satisfaction with treatment	3.20 (1.08)	93	2.8	46.8
Likelihood of recommending treatment	3.21 (1.10)	93	4.2	51.1

Two item-item pairs exhibited correlation coefficients near or greater than 0.70 (Table 3). Interference with daily activities due to treatment-related travel and overall satisfaction were correlated at r = 0.68 (*P *< 0.001). Overall satisfaction and likelihood of recommending treatment had r = 0.77 (*P *< 0.001). All 3 items were retained since the correlations were at or just above the predetermined threshold for consideration and we felt each item measured distinct aspects of treatment burden and satisfaction.

**Table 3:** Item-Item Correlation Coefficients (Spearman's rho)

**Item**	**1**	**2**	**3**	**4**	**5**	**6**	**7**	**8**	**9**	**10**
1. Demands of treatment	1									
2. Schedule flexibility	0.192*	1								
3. Difficulty in receiving every injection	0.399**	0.217**	1							
4. Treatment-related travel interference w/daily activity	0.468**	0.130	0.367**	1						
5. Overall inconvenience	0.524**	0.119	0.503**	0.682**	1					
6. Inconvenience to family/caregivers	0.377**	0.119	0.257**	0.495**	0.557**	1				
7. Overall physical discomfort from injections	0.368**	0.123	0.393**	0.313**	0.347**	0.265**	1			
8. Financial burden from out-of-pocket costs	0.404**	0.245**	0.341**	0.375**	0.369**	0.412**	0.240**	1		
9. Satisfaction with treatment	0.277**	0.245**	0.250**	0.189*	0.130	0.151	0.322**	0.228**	1	
10. Likelihood of recommending treatment	0.316**	0.168*	0.261**	0.252**	0.230**	0.213**	0.271**	0.239**	0.765**	1

*P < 0.010

**P < 0.001

### Subscale development

Two subscales were identified by factor analysis (Table 4). The first factor was composed of 7 questions that measure burden of treatment, inconvenience, and physical pain. The second factor contained 2 items capturing overall satisfaction. Internal consistency for each subscale is 0.83 (*P *< 0.001). Test-retest reliability was moderate between weeks 5 and 9 in patients with stable MOS SF-36 Global Health scores (128/284 participants). The item ("relating to difficulty in receiving every injection") did not load strongly on either subscale and was excluded from further analyses.

**Table 4:** Factor Scores and Subscales, Internal Consistency, and Inter-Rater Reliability

	**Subscale**	
**Item**	**Inconvenience **(n = 266)	**Satisfaction **(n = 265)
Demands of treatment	**0.595**	0.103
Schedule flexibility	**0.600**	0.094
Difficulty in receiving every injection	0.057	0.241
Treatment-related travel interference w/daily activity	**0.710**	-0.007
Overall inconvenience	**0.878**	-0.110
Inconvenience to family/caregivers	**0.714**	-0.038
Overall physical discomfort from injections	**0.451**	0.187
Financial burden from out-of-pocket costs	**0.507**	0.038
Satisfaction with treatment	-0.037	**0.813**
Likelihood of recommending treatment	0.084	**0.761**
		
Cronbach's Alpha (week 5)	0.83 (*P *< 0.001)	0.83 (*P *< 0.001)
ICC (weeks 5–9), patients with stable MOS Global Health	0.67 (*P *= 0.210)	0.45 (*P *= 0.020)

Note: Components of each subscale are denoted in bold.

### Construct validity

The Inconvenience subscale correlated negatively with descriptive questions on resources devoted to treatment (Table 5, r = -0.19 to -0.61). The Satisfaction subscale correlated positively with MOS SF-36 Global Health (r = 0.13 to 0.25) and negatively with resources devoted to treatment (r = -0.22 to -0.28). These relationships were largely as hypothesized. However, neither subscale correlated significantly with Hb levels at either week 5 or 9. These results are consistent using both Pearson's correlation coefficients and Spearman's rank-based correlation coefficients.

**Table 5:** Correlation Coefficients between Each PSQ-An Subscale and other Measure Scores

	**Subscale**							
	**Inconvenience (n = 266)**				**Satisfaction (n = 265)**			
	Week 5		Week 9		Week 5		Week 9	
**Item**	Pearson	Spearman	Pearson	Spearman	Pearson	Spearman	Pearson	Spearman

General Health	0.096	0.135	0.123	0.141	0.224*	0.248**	0.176*	0.133*
Hb level	-0.043	-0.065	-0.112	-0.079	0.033	0.040	0.072	0.038
								
Resources devoted to injections for anemia treatment during the past 4 weeks:								
Number of office visits	-0.047	-0.028	-0.164	-0.087	-0.062	-0.049	-0.018	0.012
Time spent traveling for office visits for each injection	-0.153	-0.188*	-0.203	-0.223*	-0.078	-0.150	0.001	-0.048
Time spent at the office to review your injection	-0.195*	-0.135	-0.314**	-0.245	-0.260**	-0.228*	-0.149	-0.231*
Number of times family/friends/caregiver inconvenienced	-0.527**	-0.514**	-0.361**	-0.607**	-0.191	-0.249**	-0.123	-0.137
Out-of-pocket expenses related to injections	-0.264**	-0.276**	-0.148	-0.380**	-0.000	-0.049	0.035	-0.012
Number of times schedule was rearranged for office visits	-0.186	-0.476**	-0.545**	-0.556**	0.004	-0.066	-0.074	-0.113
Hours of work missed due to injections	-0.181	-0.351**	-0.219	-0.316	0.004	-0.076	-0.095	-0.072
Time activities of daily living reduced due to injections	-0.324**	-0.522**	-0.394**	-0.535**	-0.158	-0.280**	-0.073	-0.176
About how many hours did caregivers miss from work	-0.367**	-0.442**	-0.240	-0.431**	-0.049	-0.103	0.039	-0.082

*P < 0.010, **P < 0.001

### Test of known-groups discriminant validity

The 2 PSQ-An subscales correlated moderately with MOS SF-36 Global Health score, Hb level, and ECOG scores (Table 6). ANOVA showed that only the Satisfaction subscale had a significant (*P *= 0.006) relationship with Global Health.

**Table 6:** Results of ANOVA for known group discriminant validity

		**PSQ-An Subscale**					
		**Inconvenience**			**Satisfaction**		
**Item**	**Response Category**	n	Mean (SD)	*P *value	n	Mean (SD)	*P *value
Self-Reported General Health	Poor	22	3.06 (0.95)	0.13	22	2.95 (1.14)	0.006
	Fair	108	3.24 (0.64)		107	2.99 (1.03)	
	Good	93	3.37 (0.58)		93	3.35 (0.92)	
	Very Good	27	3.47 (0.71)		27	3.53 (0.81)	
	Excellent	12	3.12 (0.99)		12	3.67 (0.58)	
Hb level (CTC)	>12.0 g/dL (female); >14.0 g/dL (male)	27	3.26 (0.68)	0.17	26	3.25 (0.95)	0.83
	10.0 – 12.0 g/dL (female); 10.0 – 14.0 g/dL (male)	104	3.23 (0.71)		104	3.04 (1.07)	
	8.0 – 10.0 g/dL	20	3.57 (0.30)		20	3.10 (1.00)	
	<8.0 g/dL	2	3.64 (0.51)		2	3.00 (1.41)	
ECOG Score*	0. Fully active, able to carry on all pre-disease performance without restriction	151	3.26 (0.72)	0.58	151	3.20 (1.00)	0.18
	1. Restricted in physically strenuous activity but ambulatory and able to carry out work of a light or sedentary nature, eg, light house work, office work	101	3.34 (0.63)		100	3.09 (1.03)	
	2. Ambulatory and capable of all self care but unable to carry out any work activities. Up and about more than 50% of waking hours	14	3.17 (0.70)		14	3.61 (0.68)	

* The conversion between Karnofsky and ECOG performance status ratings may be found at

### Effect size and responsiveness

The effect size between week 5 and week 9 for the Satisfaction subscale was 0.44 (Table 7). This is a moderately large detectable change over this period. In contrast, the effect size for the Inconvenience subscale was a moderate 0.13. Changes over this period in the Satisfaction subscale also correlated with changes in MOS SF-36 Global Health over this period (Table 8). The trends in changes in the Inconvenience subscale scores and in changes in MOS SF-36 Global Health were not statistically significant.

**Table 7:** Subscale Effect Sizes at Week 5 and Week 9 for Patients with Improved MOS Global Health

	**PSA-An Subscale**			
	**Inconvenience**		**Satisfaction**	
**Time Period**	*n*	*mean (SD)*	*n*	*mean (SD)*
Week 5	266	3.26 (0.63)	265	2.95 (1.11)
Week 9	223	3.18 (0.64)	222	3.35 (0.62)
				
Effect Size week 5–9	0.13		0.44	

**Table 8:** ANOVA for Week 5-Week 9 Differences in Subscales by MOS Global Health

	**Change in Subscale**			
	**Inconvenience**		**Satisfaction**	
**Change in MOS Global Health**	*n*	*mean (SD)*	*n*	*mean (SD)*
1 (*decrease*)	36	-0.011 (0.56)	36	-0.042 (0.80)
2 (*no change*)	128	-0.038 (0.51)	127	0.063 (0.90)
3 (*improvement*)	44	-0.078 (0.66)	43	0.407 (1.08)
				
F	0.16		2.87	
Pr > F	0.850		0.059	

## Discussion

The results from this study support the validity and reliability of the scale part of the Patient Satisfaction Questionnaire for Anemia Treatment (PSQ-An) for measuring satisfaction with anemia injection treatment for cancer patients. Item-item correlations were moderate and suggested that the individual question items measured distinct constructs. There were moderate ceiling effects on several component question items, perhaps reflecting difficulty in measuring high levels of satisfaction. This effect lowered variance in the PSQ-An subscales, which in turn may have led to the moderate results seen in some of the validity tests. For example, the moderate trends noted in convergent, divergent, and known-group discriminant validity may have been due, in part, to limited variation in the subscales. With 40% or more patients reporting high satisfaction on most question items, the potential strength of trends with independent measure of health status are in turn reduced.

Items loaded distinctly onto the 2 subscales and internal consistency of both the Inconvenience and Satisfaction subscales were high. Despite including only patients with stable MOS SF-36 Global Health for the test-retest assessment, the subscale scores of the PSQ-An had moderate reproducibility over a 4-week test-retest timeframe (ICC = 0.45 for Satisfaction and 0.67 for Inconvenience). These results were not unexpected given the potential improvement in anemia over 4 weeks of treatment. This suggests that overall satisfaction with treatment may change substantially over the 4-week study period even when controlling for overall health status. Indeed, the effect size for the Satisfaction subscale shows that patients' value assessments of the treatment underwent large changes over the study period.

This study has several limitations. First, this study included primarily female patients due to the inclusion criteria, so further evaluation of the PSQ-An is warranted prior to use in other patient populations. Second, the initial item pool was drawn from the literature, not developed from patient focus groups. Nor were these items cognitively tested in patient focus groups. Input from patients may have revealed additional concepts of satisfaction not incorporated into current literature on which our item pool was based. Cognitive testing or debriefing may also have improved the wording/content of the questionnaire; for example, it may have identified better response scales with less potential for ceiling effects. Third, we did not stratify our validation and analysis by disease stage. It is possible that patients with stage IV cancers (48% of our sample) respond quite differently to treatment satisfaction questions than do patients with lesser progression. Again, extrapolations to other patient populations should be made cautiously. Finally, while it is unlikely that the observed ceiling effects are due to response bias, in its present form the PSQ-An may not fully capture the range of satisfaction cancer patients can express about anemia treatment. Consideration could be given to additional response categories to encompass a broader spectrum of satisfaction responses.

The Patient Satisfaction Questionnaire for Anemia Treatment (PSQ-An) is a validated, treatment-specific instrument for measuring satisfaction with anemia treatment for cancer patients. The 2 subscales of the PSQ-An reflect the burden of injection anemia treatment on cancer patients and their assessment of the overall value of that treatment. This instrument has potential to aid clinicians in their understanding of the various aspects of patient satisfaction with anemia treatment and allow clinicians to optimize patient care.

## Competing interests

Robert J. Nordyke is employed by Cerner Health Insights, which provides consulting services to Amgen, Inc. Chiun-Fang Chiou, Joel F. Wallace, and Bin Yao are employed by Amgen, Inc.

## Authors' contributions

Robert J. Nordyke: contributed to design, analysis and interpretation of data; drafted the manuscript; revised manuscript for important content; gave final approval of manuscript

Chih-Hung Chang: contributed to design, analysis and interpretation of data; drafted the manuscript; revised manuscript for important content; gave final approval of manuscript

Chiun-Fang Chiou: contributed to design, analysis and interpretation of data; revised manuscript for important content; gave final approval of manuscript

Joel F. Wallace: contributed to design, collection, analysis and interpretation of data; revised manuscript for important content; gave final approval of manuscript

Bin Yao: contributed to design, collection, analysis and interpretation of data; revised manuscript for important content; gave final approval of manuscript

Lee S. Schwartzberg: contributed to the interpretation of data; revised manuscript for important content; gave final approval of manuscript

## Supplementary Material

Additional File 1 Nordyke additional. Appendix: Patient Satisfaction Questionnaire for Anemia Injection Treatment (PSQ-An). Click here for file
