# Supplementary material for: Validation of a patient satisfaction questionnaire for anemia treatment, the PSQ-An
Source: Health Qual Life Outcomes. 2006 May 3;4:28. doi: 10.1186/1477-7525-4-28 (PMC1526422; doi:10.1186/1477-7525-4-28)
Supplement: Additional File 1 — Nordyke additional. Appendix: Patient Satisfaction Questionnaire for Anemia Injection Treatment (PSQ-An). [file 1477-7525-4-28-S1.doc]

**APPENDIX: Patient Satisfaction Questionnaire for Anemia Injection Treatment**

**Resources Devoted to Treatment Questions.**

***Please respond by writing your answer in the space provided*.**

- How many times did you go to the doctor’s office or clinic to receive your injections for anemia treatment during the past 4 weeks? *(# times)*
- How much time, on average, did you spend traveling to and from the doctor’s office or clinic for each of your injections for anemia treatment during the past 4 weeks? *(Hours and minutes)*
- How much time, on an average, did you spend at the doctor’s office or clinic to review your injection for anemia treatment during the past 4 weeks? *(Hours and minutes)*

Please provide a breakdown of this time spent at the doctor’s office or clinic for the following:

- Check-in time: *(Hours and minutes)*
- Waiting time: *(Hours and minutes)*
- Injection time: *(Hours and minutes)*
- Other (please specify): *(Hours and minutes)*
- How many times were your family, friends and/or caregiver inconvenienced by your need to get injections for anemia treatment during the past 4 weeks? (# times)
- How much did you spend in out-of-pocket expenses related to injections for anemia treatment during the past 4 weeks (include the money you spent on physician visits, medications, transportation, and hired help to assist you with doctor’s office or clinic visits)? ($)
- How many times did you have to rearrange your schedule for medical visits to receive injections for anemia treatment during the past 4 weeks? (# times)
- How many hours did you miss from work because of you injections for anemia treatment during the past 4 weeks (Include hours that you missed due to sick days, going in late, leaving early, etc…)? If you do not work fill in the blank with N/A. (Hours)
- How much time did you have to cut down on activities of daily living because of your injections for anemia treatment during the past 4 weeks? (Hours and minutes)
- In your opinion, how many hours did your caregivers miss from work because of your injections for anemia treatment during the past 4 weeks (Include hours they missed due to going in late, leaving early, etc…)? If they (caregivers) do no work fill in the blank with N/A. (Hours)

**Treatment Burden and Satisfaction Questions**

***Please respond by checking one box for each question. (All responses are: Not At All/Mildly/Somewhat/Moderately/Extremely)***

***During the past 4 Weeks…***

How demanding were your injections for anemia treatment (in terms of time, effort, etc…)?

How flexible was the schedule to get your injections for anemia treatment? Did you find it difficult to receive every injection for anemia treatment your doctor recommended?

How much did traveling to the doctor’s office or clinic to receive your injections for anemia treatment interfere with your usual daily activities?

Overall, how much were you inconvenienced by your need to receive your injections for anemia treatment?

In your opinion, how inconvenienced were your family, friends and/or caregivers by your need to get injections for anemia treatment?

Overall, how painful or physically uncomfortable were your injections for anemia treatment?

How financially strained did you feel because of the out-of-pocket costs of your injections for anemia treatment?

How satisfied were you with your injections for anemia treatment?

How likely are you to recommend injections for anemia treatment to a friend or family member who may need them?
